# When Risk Management Delays Recovery: A Service Evaluation of Acute Admissions in Emotionally Unstable Personality Disorder

**DOI:** 10.1192/bjo.2026.11596

**Published:** 2026-06-30

**Authors:** Gayathri Rangith

**Affiliations:** Cygnet Health Care, Oldbury, United Kingdom

## Abstract

**Aims:**

Patients with Emotionally Unstable Personality Disorder (EUPD) are frequently admitted to acute psychiatric wards during periods of crisis. However, concerns have been raised regarding prolonged admissions, delayed discharge, and potential iatrogenic effects of high-containment care. This service evaluation aimed to compare length of stay (LOS), observation requirements, and discharge outcomes for patients with EUPD versus non-EUPD patients admitted to an acute female ward, and to explore factors contributing to prolonged admission.

**Methods:**

A retrospective service evaluation was conducted of all admissions to a 15-bed acute female psychiatric ward over a 12-month period. Patients with an ICD-10 diagnosis of EUPD (n=19) were compared with non-EUPD admissions (n=54). Data were extracted fromclinical records, incident reports, and discharge documentation, including LOS, observation levels, incident patterns, and placement outcomes. One fully anonymised illustrative vignette was included to demonstrate a commonly observed clinical trajectory. No patient-identifiable data were collected.

**Results:**

Patients with EUPD demonstrated substantially longer admissions than non-EUPD patients, with a median LOS of approximately six months (range 3–18 months), compared with 4–8 weeks in the non-EUPD cohort. Nearly all EUPD patients required enhanced observations, including one-to-one nursing, at some point during admission, often recurrently. Attempts to reduce observation levels were frequently followed by behavioural escalation, particularly during periods of planned discharge or placement identification. Placement pathways were commonly delayed or disrupted due to ongoing risk behaviours, high observation requirements, or withdrawal of placement offers following incidents. These patterns were consistently observed across the EUPD cohort and contrasted with shorter, more linear admission pathways in non-EUPD patients.

**Conclusion:**

Prolonged acute inpatient admissions among patients with EUPD appear to be driven less by illness severity and more by systemic and environmental factors within high-containment settings. Risk-averse observation practices may unintentionally reinforce dependency, contribute to behavioural escalation around transitions of care, and impede discharge planning. These findings highlight the need for alternative models of crisis support, clearer step-down strategies, and greater caution in interpreting functional assessments conducted within acute inpatient environments.

